# Caries and Restoration Detection Using Bitewing Film Based on Transfer Learning with CNNs

**DOI:** 10.3390/s21134613

**Published:** 2021-07-05

**Authors:** Yi-Cheng Mao, Tsung-Yi Chen, He-Sheng Chou, Szu-Yin Lin, Sheng-Yu Liu, Yu-An Chen, Yu-Lin Liu, Chiung-An Chen, Yen-Cheng Huang, Shih-Lun Chen, Chun-Wei Li, Patricia Angela R. Abu, Wei-Yuan Chiang

**Affiliations:** 1 Department of General Dentistry, Chang Gung Memorial Hospital, Taoyuan City 33305, Taiwan; louiszzzzz@cgmh.org.tw (Y.-C.M.); mr2005@cgmh.org.tw (Y.-C.H.); harrythebold@cgmh.org.tw (C.-W.L.); 2 Department of Electronic Engineering, Chung Yuan Christian University, Taoyuan City 32023, Taiwan; g10976016@cycu.edu.tw (T.-Y.C.); g10776018@cycu.edu.tw (H.-S.C.); s10726334@cycu.edu.tw (S.-Y.L.); s10726325@cycu.edu.tw (Y.-A.C.); s10726335@cycu.edu.tw (Y.-L.L.); 3 Department of Computer Science and Information Engineering, National Ilan University, Yilan City 26047, Taiwan; 4 Department of Electrical Engineering, Ming Chi University of Technology, New Taipei City 243303, Taiwan; 5 Department of Information Systems and Computer Science, Ateneo de Manila University, Quezon City 1108, Philippines; pabu@ateneo.edu; 6 National Synchrotron Radiation Research Center, Hsinchu City 30076, Taiwan; chiang.wy@nsrrc.org.tw

**Keywords:** biomedical image, bitewing film, Gaussian high-pass filter, Otsu’s thresholding, deep learning, CNN, transfer learning, AlexNet

## Abstract

Caries is a dental disease caused by bacterial infection. If the cause of the caries is detected early, the treatment will be relatively easy, which in turn prevents caries from spreading. The current common procedure of dentists is to first perform radiographic examination on the patient and mark the lesions manually. However, the work of judging lesions and markings requires professional experience and is very time-consuming and repetitive. Taking advantage of the rapid development of artificial intelligence imaging research and technical methods will help dentists make accurate markings and improve medical treatments. It can also shorten the judgment time of professionals. In addition to the use of Gaussian high-pass filter and Otsu’s threshold image enhancement technology, this research solves the problem that the original cutting technology cannot extract certain single teeth, and it proposes a caries and lesions area analysis model based on convolutional neural networks (CNN), which can identify caries and restorations from the bitewing images. Moreover, it provides dentists with more accurate objective judgment data to achieve the purpose of automatic diagnosis and treatment planning as a technology for assisting precision medicine. A standardized database established following a defined set of steps is also proposed in this study. There are three main steps to generate the image of a single tooth from a bitewing image, which can increase the accuracy of the analysis model. The steps include (1) preprocessing of the dental image to obtain a high-quality binarization, (2) a dental image cropping procedure to obtain individually separated tooth samples, and (3) a dental image masking step which masks the fine broken teeth from the sample and enhances the quality of the training. Among the current four common neural networks, namely, AlexNet, GoogleNet, Vgg19, and ResNet50, experimental results show that the proposed AlexNet model in this study for restoration and caries judgments has an accuracy as high as 95.56% and 90.30%, respectively. These are promising results that lead to the possibility of developing an automatic judgment method of bitewing film.

## 1. Introduction

Caries screening and detection is one of the most frequent operations in daily dental practice. Detection of caries in its early stage can prevent patients from accepting further invasive treatment procedures. Caries lesions have traditionally been diagnosed via visual–tactile detection inspection in combination with bitewing radiography. Moreover, bitewing radiography has long been used by dentists for the detection of proximal caries [1] that are clinically hidden from a careful clinical visual examination [2]. The recommendation for a posterior bitewing examination is that it should capture an image of the crowns of the teeth from the distal surface of the canine to the distal surface of the most posterior erupted molar [3]. The importance of radiographic examination to diagnose caries lesions in proximal surfaces of the teeth is well established [4]. It is an inexpensive and easy-to-use method that is commonly employed in everyday dental practice to support clinical findings. In addition to caries lesions, bitewing radiography may also offer other information such as restorations [5] and bony structures. However, interpreting the radiographic appearance of caries lesions in bitewing radiography can sometimes be subjective. Interpretation of bitewing radiography can be time-consuming for dentists in their daily dental practice, and different examiners often have different interpretations.

Recently, artificial intelligence (AI) and deep learning have been well developed and have evolved. Using big data analysis and machine learning as auxiliary tools in medicine is a trend. For example, [6] proposed an intelligent medicine recognition method, which can be converted into the largest character recognition in the picture; [7] focused on how machine learning can help in the association with disease risk; [8] developed a machine learning model with decision stumps as a base learner with different feature combinations and preprocessing procedures; [9] developed predictive models using four machine learning methods (support vector machine (SVM), least squares support vector machine (LS-SVM), artificial neural networks (ANN), and random forest (RF)) to detect PC cases using available prebiopsy information. In dentistry, research on deep learning has also increased gradually [10] and has been widely applied in different specialties [11,12,13,14]. Applying deep learning to detect caries lesions and restorations in bitewing radiography could potentially save more clinical time for dentists to focus on treatment planning and clinical operations. Furthermore, AI technology can be used to classify examination results (e.g., restorations) in the database for further use, making data collection and medical document recording more efficient [15]. Most of the research on machine learning and big data analysis is related to the change in grayscale values in images. In [16], researchers designed three different models on two different architectures to classify three common diseases: dental caries, periapical infection, and periodontitis. It was found that transfer learning with the VGG16 pretrained model achieved better accuracy. A small dataset consisting of 251 RVG X-ray images was used for training and testing purposes. Experimental results for different models were discussed and showed an achieved overall accuracy of 88.46%. Furthermore, Casalegno et al. [17] combined near-infrared transillumination (TI) imaging with CNN to achieve the detection purpose by analyzing dental images. On the other hand, Aberin and de Goma [18] researched the detection of periodontal disease. The methodology of this research dwelt more on classifying the microscopic dental plaque images fed into the neural networks as healthy or unhealthy. This study used a convolutional neural network as the classifier and utilized the AlexNet architecture to classify the images using Tensorflow, yielding an accuracy rate of 75.5% and a mean square error of 0.05348436995. In another study, Chen et al. [19] used a faster region-based CNN (faster R-CNN) to detect and number periodontal ligament teeth. A filtering algorithm was used to delete overlapping boxes detected with the same tooth. Other models were used to detect missing teeth. Lastly, a rule-based module was proposed to match the label of the detected tooth frame to modify any detection result that violated some intuitive rules. In the study in Liu et al. [20], an automatic diagnosis model trained by MASK R-CNN was developed for the detection and classification of seven different dental diseases including decayed tooth, dental plaque, fluorosis, and periodontal disease, with a diagnosis accuracy of up to 90% along with high sensitivity and high specificity. The study in Moran et al. [21] proposed a convolutional neural network to classify periodontal bone destruction in periapical radiographs. This study considered 1079 interproximal regions extracted from 467 periapical radiographs. These data were annotated by experts and used to train a ResNet and an Inception model, which were evaluated with a test set. The Inception model presented the best results and an impressive rate of correctness even on a small and unbalanced dataset. The final accuracy, precision, recall, specificity, and negative predictive values were 0.817, 0.762, 0.923, 0.711, and 0.902, respectively. In previous research, it can be seen that a convolutional neural network (CNN) is a deep learning method which is most often used to analyze visual images. The research in this study aims to establish two CNN models through transfer learning to classify caries and restorations, to establish a tooth segmentation system, and to build a database to provide the CNN models with images for training and verification. Transfer learning means the use of neural networks originally designed for another task in a new field.

This study proposes a caries and lesion area analysis model based on CNN with transfer learning. This model can analyze cavities and prosthodontics in bitewing radiography and provide dentists with a more accurate objective judgment of the data to achieve the goal of automating diagnosis and treatment. In this study, AlexNet is used as the basis of the CNN model, and its hyperparameters are modified to achieve the desired classification results. The AlexNet model contained eight layers; the first five layers were convolutional layers, some of them followed by max-pooling layers, and the last three layers were fully connected layers. The non-saturating ReLU activation function was used, which showed improved training performance over tanh and sigmoid. Moreover, a standardized database established through a set of steps is also proposed in this study. The procedure includes three main steps to convert the bitewing image into samples of a single tooth per image, which can increase the accuracy of the model. This was combined with data enhancement technology [17], including the use of flip, zoom, rotation, translation, contrast, and brightness to increase the amount of data, as well as vertical flip and horizontal flip processing, which relieves the pressure on collecting clinical datasets for training AI models. The first step is the preprocessing of the original dental image. Since X-ray images are quite close to light and dark pixels, this study first applied Gaussian high-pass filter processing to the images, before passing them through an iterative threshold operation to obtain a high-quality binarization. The second step is the dental image segmentation procedure, from development of the cutting method to the conversion of the dental X-ray image into separate individual tooth samples. The third step is dental image masking, which masks the fine broken teeth in the sample, thereby enhancing the quality of the training.

During the treatment of tooth decay, if the cause of the problem is detected early, the treatment of findings will be relatively easy, thus preventing the caries from spreading. Therefore, early detection of the disease is very important and necessary. The analysis method of dental caries and restorations in bitewing radiography proposed in this study can provide dentists with more accurate objective judgment data, so as to achieve the purpose of developing automatic diagnosis and treatment planning as a technology to assist precision medicine. The proposed method not only reduces the workload of dentists, but also allows them to have more time for professional clinical treatment, improves the quality of medical resources, and achieves the goal of a harmonious relationship between doctors and patients.

The introductory structure of this study is followed by an introduction of the materials and methods used for the caries and lesion area analysis model based on a convolutional neural network (CNN). In the third section, the evaluation methods of the model and the experimental results are presented and analyzed. Then, the findings are discussed in the fourth section. Lastly, the fifth section presents the conclusions and future perspectives.

## 2. Materials and Methods

**Figure 1:**
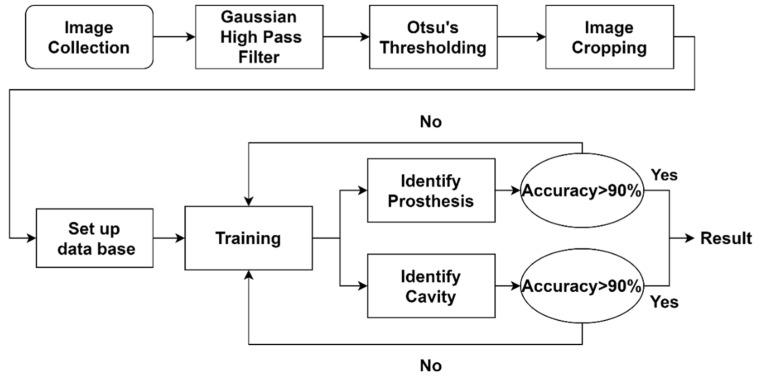
Flowchart of the proposed method.

The purpose of this study was to develop a CNN model for transfer learning to identify and classify restoration and caries findings given a bitewing image. The proposed method is divided into four steps: (1) image preprocessing, (2) image cropping, (3) setting up the database, and (4) CNN image identification. Detailed research steps, as shown in Figure 1.

### 2.1. Image Preprocessing

The objective of this step is to successfully binarize the image and to clearly separate the target of interest from the background. The success of this step can greatly affect the cutting judgment in the image cropping step. According to the clinical collection of bitewings, the image can be divided into three parts: the background, with the lowest pixel value (close to 0), the alveolar bone, with an averaged pixel value, the teeth, with the highest pixel value (above 120). The advantage of the binarization step is that it can effectively emphasize the details in the image that are not easily discovered, thus enabling the separation of the area of interest. After the bitewing image is binarized, the pixel value of its background is changed to the minimum value of a grayscale image (i.e., 0) while the pixel values of the teeth and alveolar bones are changed to the maximum value (i.e., 255).

If the bitewing image threshold value is selected directly, the result is not as expected. Taking into consideration the study in Nomir and Abdel-Mottaleb [22] as an example, after the use of iterative thresholding, direct binarization is done. This method cannot actually separate the target area of interest from the background. This is due to each image not necessarily being divisible into the abovementioned three parts, instead being independent of each other. Various parts of the image may appear to have uneven grayscale values. In this case, the target area of interest cannot be accurately separated from the background, thus resulting in an incomplete tooth image. Encountering such a case can significantly affect the subsequent image cropping step and incorrectly determine the cutting line. Accordingly, this study first used a Gaussian high-pass filter and then Otsu’s algorithm to select the threshold and avoid the problems encountered by previously proposed methods in the literature.

#### 2.1.1. Gaussian High-Pass Filter

The Gaussian high-pass filter is based on frequency domain filtering. The ideal formula is shown in Equation (1), where $D_{0}$ is the cutoff frequency. One benefit of using frequency domain filtering is that some enhancement tasks that are difficult to express in the spatial domain become simpler and more intuitive in the frequency domain [23].

(1) $$H\left(u,v\right)=\{\begin{array}{cc} 1 & if\;D\left(u,v\right)\leq D_{0}\\ 0 & if\;D\left(u,v\right)\geq D_{0} \end{array}.$$

The Gaussian high-pass filter is now explored, which enables sharpening of the image to better extract the edge information in the image. The formula is shown in Equation (2), where the image size is u×v.

(2) $$H\left(u,v\right)=1-e^{\frac{-D{\left(u,v\right)}^{2}}{2\times D_{0}}}.$$

A smaller value of $D_{0}^{}$ denotes less accurate edge feature extraction, which contains more non-edge information. A larger value of $D_{0}^{}$ denotes more accurate edge feature extraction; however, incomplete edge information may arise. Therefore, according to the need of having different $D_{0}$ values, the value of the data also varies. In this study, the bitewing image was sharpened by applying filtering. The filtered image highlights impurities and edges, and then the filtered image is subtracted from the original image to remove impurities from the original image. The difference in grayscale values between the different parts of the image is then made evident [24].

#### 2.1.2. Otsu’s Thresholding

**Figure 2:**
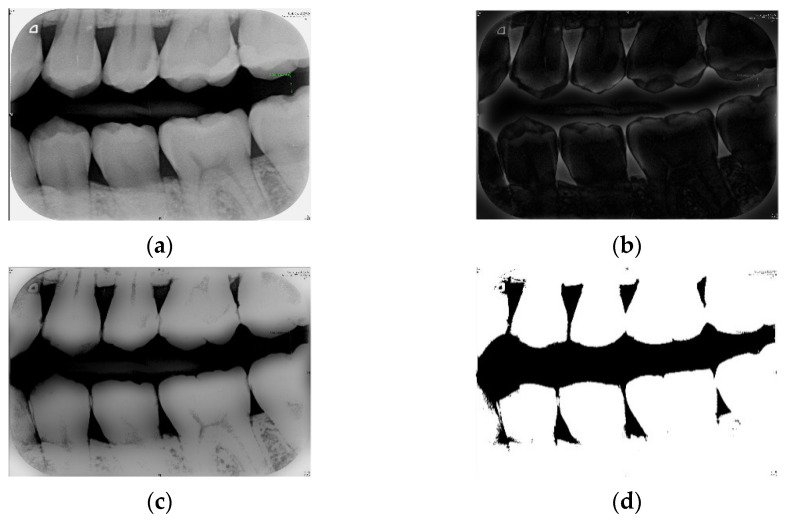
(**a**) The bitewing film; (**b**) the filtered result of (**a**) using the Gaussian high-pass filter; (**c**) the result of (**a**) minus (**b**); (**d**) the result of (**c**) after the binarization.

In this study, Otsu’s algorithm was adopted to select the threshold value. Its principle is to automatically find the threshold value for cluster-based images. The algorithm assumes that the image has a two-mode histogram (a histogram distinguished by foreground and background pixels). The optimal threshold that can separate the foreground and background pixels can be calculated by using the exhaust method in order to obtain the minimum number of variations in individual classes and the maximum number of between-class variations. According to the histogram obtained from different thresholds, the corresponding number of individual class and between-class variations is obtained, and the differences are compared [25,26]. The implementations are shown in Figure 2.

### 2.2. Image Cropping and Masking

In order for the CNN model to judge whether there are findings on both sides of each tooth in the bitewing film, each tooth in the bitewing film must be identified and separated into individual photos. In this study, in order to separate each tooth in the bitewing image, first, horizontal projection [22] was applied to separate the upper and lower rows of teeth in the bitewing film into two photos, while vertical projection [22] was applied to separate the individual teeth from the upper and lower rows of teeth into a single individual tooth photo. To present a complete image in the output photo of a single tooth after slicing, the output photo must select the largest range. However, selecting the largest range can include images that cause the model to misjudge the data. As such, it is essential to mask the images that can cause misjudgment. Lastly, the photo is sliced in half to display the left and right sides of the tooth, which are used as input to the CNN model for judgment.

#### 2.2.1. Horizontal Projection

**Figure 3:**
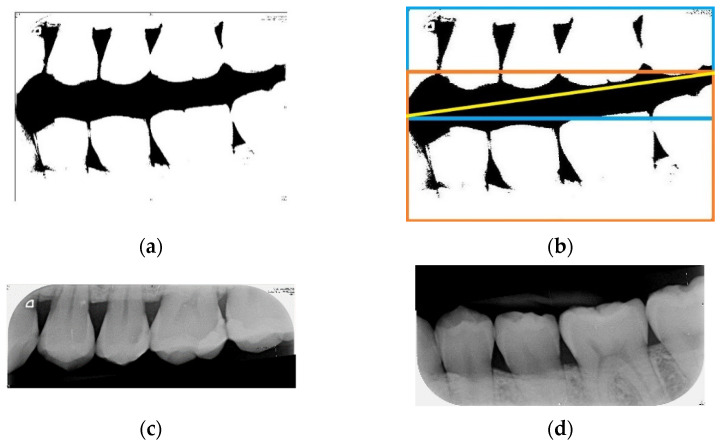
(**a**) The binarized bitewing film; (**b**) the schematic diagram of slicing the bitewing film, where the yellow line is the separating line, the blue bounding box is the area of the photo of the upper row of teeth in the bitewing film, and the orange bounding box is the area of the photo of the lower row of teeth in the bitewing film; (**c**,**d**) photos of the upper and lower rows of teeth after slicing, respectively.

The goal of horizontal projection is to slice the bitewing film into photos of the upper and lower rows of teeth. Furthermore, the method of horizontal projection involves adding the pixel values of each row of the binarized photo, followed by identifying the row with the smallest total value as the separating line. The formula is shown in Equation (3). The principle involves image processing of the bitewing film where the background between the upper and lower teeth is binarized to zero, whereby adding the pixel values of the row between the upper and lower teeth is smallest. According to Figure 3a, a horizontal line cannot be used to precisely separate the upper and lower rows of teeth.

Therefore, to find an ideal separating line, there is a need to rotate the photo first. A horizontal projection is subsequently performed every time the photo is rotated by 1°. The minimum total value found from each horizontal projection is then recorded. Next, the smallest value and its corresponding angle in the record are identified, and the photo is then rotated to this angle to perform horizontal projection. The separating line found at this time is the most ideal one. The formula is shown in Equation (4). Using this ideal separating line, the bitewing film can be sliced into photos of the upper and lower rows of teeth, as shown in Figure 3b.

(3) $$H\left(i\right)=\overset{m}{\underset{j=1}{\sum }}f\left(i,j\right).$$

The formula of horizontal projection is presented above; let *f*(*i*, *j*) be the *m* × *n* binary image.

(4) $$\left(\theta ,\;y\right)=\arg\min_{\left(\theta ,y\right)}H^{\theta }\left(y\right),$$

where $H^{\theta }\left(y\right)$ is the horizontal integral projection obtained by rotating the binary image by angle θ.

#### 2.2.2. Vertical Projection

**Figure 4:**
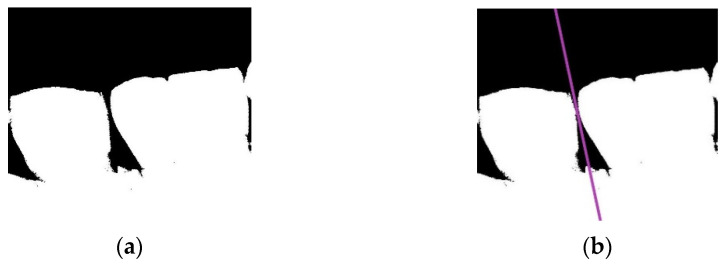
(**a**) The binarized photo showing two teeth; (**b**) precise separating line (purple), determined by rotating the photo and performing vertical projection.

The goal of vertical projection is to slice the photo of the upper row of teeth or lower row of teeth into a single tooth photo. Vertical projection is similar to horizontal projection. The main difference is that the method of vertical projection adds the pixel values of each column in the binarized photo, and then the column with the smallest total value is identified as the separating line. The formula to implement this step is shown in Equation (5). According to Figure 4a, using a vertical line cannot precisely separate the adjacent teeth. Therefore, to present a complete image of the output photo after slicing, a more precise separating line must be determined.

This method is similar to finding the line separating the upper and lower rows of teeth. First, the photo is rotated, and then vertical projection is performed. The minimum value obtained from vertical projection is recorded each time the photo is rotated by 1°. Then, the minimum value and the corresponding photo rotation angle in the recorded data are determined. The photo is then rotated to the angle determined in the previous step, before performing vertical projection to generate the precise separating line. The formula is shown in Equation (6).

(5) $$V\left(j\right)=\overset{m}{\underset{i=1}{\sum }}f\left(i,j\right).$$

The formula of vertical projection is presented above; let *f*(*i*, *j*) be the *m* × *n* binary image.

(6) $$\left(\theta ,\;x\right)=\arg\min_{\left(\theta ,x\right)}V^{\theta }\left(x\right),$$

where $V^{\theta }\left(y\right)$ is the horizontal integral projection obtained by rotating the binary image by an angle θ.

#### 2.2.3. Masking Image of Single Tooth That Can Lead to CNN Model Misjudgment

**Figure 5:**
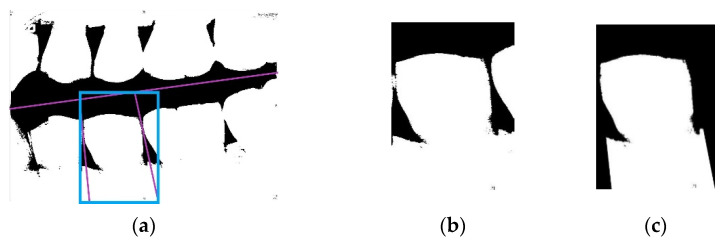
(**a**) A sample image misjudged by the CNN model with the three purple lines identified as the separating lines and the blue box as the area of the segmented tooth image after slicing; (**b**) sample photo of a single tooth without masking; (**c**) sample photo of a single tooth after masking the image misjudged by the CNN model.

Figure 5a illustrates an example of a single tooth misjudged by the CNN model. The three purple lines are the separating lines found by horizontal and vertical projection. To completely isolate the image of the tooth within the three separating lines, the area within the blue bounding box must be selected as the slicing range. However, in the blue box, only the images framed by the three separating lines are considered. Including the outer parts of the image, i.e., outside and beyond the bounding purple lines, can cause errors in the judgment of the CNN model. Therefore, images similar to this must be masked to avoid misjudgments by the model, by setting the pixel values of the rest of the image to zero.

**Figure 6:**
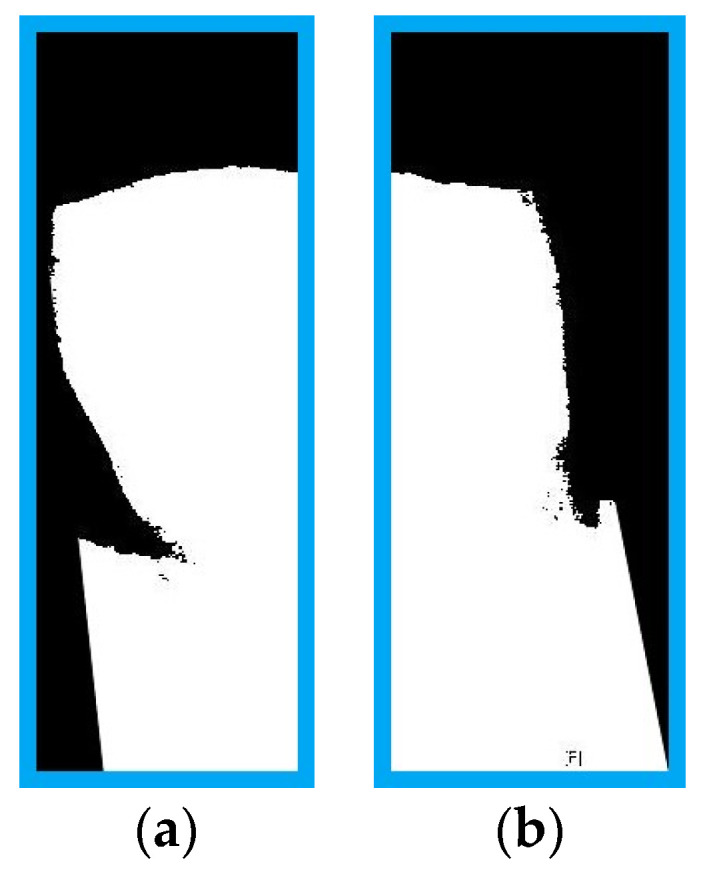
Photo of a single tooth sliced into photos of its (**a**) the right side and (**b**) the left side.

In order for the CNN model to determine whether there are caries or restorations on both sides of the tooth, the tooth needs to be cut in half to output photos of its left and right sides, as shown in Figure 6.

### 2.3. Database Setup

Clinical images were annotated by three professional dentists. All experts were employed in specialized clinics to perform operations, including caries detection, and had at least 3 years of clinical experience. The experts guided the researchers, provided knowledge of the symptoms, used actual cases to teach the researchers (describing the characteristics of dental caries and restorations), and provided clinical data to calibrate the CNN model (eliminating other nontarget symptoms). In clinical medicine, a tooth may have more than one finding at the same time. Therefore, this study established an independent database based on different findings, and a solution to the problem is presented in the next section by establishing different independent disease models. In order to reduce the computational complexity of the developed algorithm, this study used unilateral teeth to judge the results. The image library annotated by the dentists was also based on unilateral teeth for marking, as described in step 2.2. A total of 278 bitewing images were cut, and 3716 images of unilateral teeth were obtained. According to the clinical database, unilateral dental images are the basis for judging restorations and caries. Due to the limited information provided, a serious imbalance was found in the image samples of the database. The number of unilateral teeth with restorations was 610, while the number of unilateral teeth with caries was only 88.

Table 1 lists the number of images per clinical disease type, with the tooth caries having a huge gap compared to the number of restorations. If this small number of images with caries was used in CNN training, the CNN model would not be able to use its advantage, whereby the learning effectiveness would not be good enough and could not correctly judge the findings. As a solution to the imbalance, according to the method of data augmentation [17], the following transformations were applied in our training pipeline: flip, zoom, rotation, translation, contrast, brightness, vertical flipping, and horizontal flipping, increasing the number of images (data augmentation) of caries to 350. As for the restorations images, 350 out of 610 were selected. This converged the ratio between the two sets of samples, thus reducing the imbalance. The individual database now had a total of 700 images, with 350 target and non-target images in each case. The data augmentation step was only used for training the CNN model. Therefore, this did not result in confusion when verifying the CNN model.

**Figure 7:**
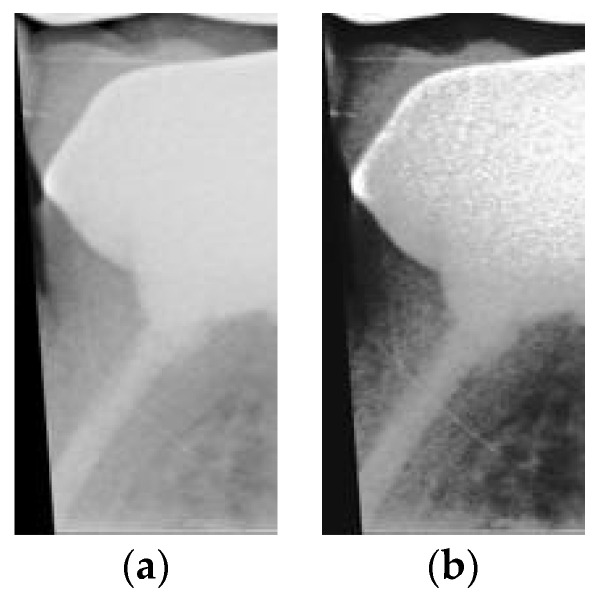
(**a**) Photo before image enhancement; (**b**) photo after image enhancement.

After image cropping, the image size was large. If we directly used the cropped images to train the model, it would cause the training of the network to be very time-consuming, and the network would have difficulty converging, thus resulting in low accuracy. Therefore, the image size within the database was standardized to 200 × 100 pixels per image at the expense of a reduction in its resolution. With this reduction in image size, the CNN could easily identify the characteristics of the image if reinforced with image processing before identification. In this study, the features of the restorations could be highlighted with contrast enhancement in order to achieve better accuracy. The image processing results are shown in Figure 7.

### 2.4. CNN Image Identification

Deep learning is a type of machine learning with artificial neural networks as the architecture. The goal is to train computers to perform human-like tasks by simulating the way in which the human brain works to achieve the same learning ability and make rapid and accurate judgments. Examples of applications include speech recognition, object identification, prediction, or even playing chess. A large and continuous amount of information needs to be provided to the computer as input to the network for training to automatically find the best function. There are many deep learning networks in the scientific community such as RNN, DNN, and CNN. Depending on the object being studied, the network selection will be different. Taking this study as an example, the aim was to judge the findings of bitewing images. Therefore, it was best to use CNN, whereby the convolution layer could be used to automatically capture the features in the image and analyze the significant characteristics for its classification and identification [27].

#### 2.4.1. Model Adjustment

This study used transfer learning to establish two networks to judge two separate findings, as discussed in detail in Section 2.3. Transfer learning is the transfer of trained models and hyperparameters to new models to help in their training. A pretrained model has learned how to recognize basic features of an image such as color, edges, and curves. On this basis, training time can be reduced, and the problems that may be encountered when developing new models can be avoided, thus increasing the efficiency of training. There are many well-known networks available on CNN, such as GoogleNet, Vgg19, ResNet50, and Alexnet. This study referred to Liawatimena et al. [28] and Oktay [29], which selected Alexnet as the main object of transfer learning, and GoogleNet, Vgg19, and ResNet50 were set up as the object of transfer learning for comparison with AlexNet. The difference in this study was a change in the input layer from 227 × 227 × 3 to 200 × 100 × 3 pixels, so as to reduce the image size after cropping. If we directly used the cropped images to train the model, the training of the network would be very time-consuming and it would have difficulty converge, thus resulting in low accuracy. Therefore, the image size within the database was standardized to 200 × 100 pixels per image at the expense of a reduction in its resolution. With this reduction in image size, the CNN could easily identify the characteristics of the image if reinforced with image processing before identification. In this study, the features of the restorations could be highlighted with contrast enhancement in order to achieve better accuracy. The results of the difference comparison between the new Alexnet and AlexNet are presented in Table 2.

Within a CNN, every layer is connected. In the case of not adjusting the stride and kernel size, additional settings are required for the fully connected layer and the calculations between the remaining layers, including the convolution layer, the ReLu layer, the normalization layer, the max-pooling layer, and the dropout layer, can be done automatically by the program. The following is a brief introduction of the AlexNet layers. (1) Convolution layer: The convolution layer is composed of parallel feature maps. New feature maps can be obtained by sliding different convolution cores on the input image and performing certain operations. (2) Normalization layer: The purpose is to increase the efficiency of training, as well as improve the efficiency (accuracy). This is the same way in which human neurons work. In addition to sending out messages, the activated neurons also inhibit their neighbors. As a result, the outgoing message noise is reduced and the signal is relatively amplified. (3) Relu layer: Also known as the linear rectifier layer, as the activation function in CNN, it can enhance the decision function in the network and the nonlinear characteristics of the whole network. (4) Pooling layer: The size of the feature map can be reduced to accelerate the training speed, but its main features can still be maintained. Parameters that need to be trained can be reduced, and the possibility of overfitting can be avoided. (5) Fully connected layer: It can also be called a classifier, in which each layer is composed of many neurons, and features are integrated through many neurons. After weight calculation, a probability is the output for each classification. As such, the specific size of the image to be modified must be selected carefully. Having too many neurons can lead to increased model complexity and easy overfitting. This also increases the calculation time and reduces efficiency. Lastly, the output layer was changed to 2 to see if the disease exists. The new Alexnet layer activations list the outputs and inputs between the layers after modification.

#### 2.4.2. Hyperparameter Adjustment

Alexnet uses a stochastic gradient descent algorithm to find the best results, based on an iterative algorithm to find the smallest value of the loss function. Each batch is trained to calculate the gradient of the loss function and update the hyperparameters, and the selection of samples in batch is done in a random manner. In this way, the value of the loss function is minimized, and the best solution is obtained. When using the algorithm, the numerical size of the updated hyperparameters is based on the learning rate. When training data, a large amount of data is fed into the network at a time, resulting in longer training time. There are memory limitations, coupled with neural networks as nonconvex functions, which in this case allows producing a local optimal solution. Therefore, the concept of mini-batch is used in this study, which takes part of the data only once for training, thereby accelerating model convergence and improving accuracy. The hyperparameter settings used in this study are shown in Table 3. There are three hyperparameters to pay attention to:

(a) LearnRate: The size of the learning rate determines whether the neural network can converge to the global minimum, i.e., to obtain a higher accuracy rate.
(b) MaxEpoch: An epoch refers to the complete passing of all training data through the neural network once.
(c) MiniBatchSize: Batch size is the minimum number of samples required for a training session.

#### 2.4.3. Training

Seventy percent of the database was used as the training set and validation set, while the remaining 30% was used as the test set. The training set included examples used for learning. The validation set was designed to obtain metrics evaluated after the network completed an epoch of training, used as the basis for adjusting the parameters. The test set was used to evaluate the performance of the model. The model was trained using the database and set hyperparameters into the network. After training, a truth table was generated after the network judged the test set. Next, it was observed whether the prediction of the network was the same as the actual result, and the effectiveness of the model was evaluated. If the results were not as expected, the hyperparameters were adjusted accordingly by finding the best value for the network.

## 3. Results

**Figure 8:**
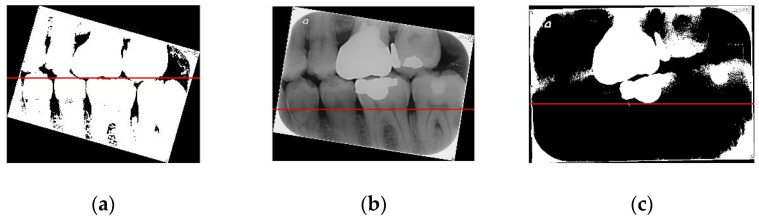
(**a**) Image processed through the Gaussian filter and Otsu’s thresholding, where the red line separates the upper and lower teeth; (**b**) image without any processing, where the red line separating the upper and lower teeth is very imprecise; (**c**) image processed only through Otsu’s thresholding, where the red line separating the upper and lower teeth is also imprecise.

Figure 8 shows the importance of image preprocessing through the Gaussian filter and Otsu’s thresholding for image cropping.

**Figure 9:**
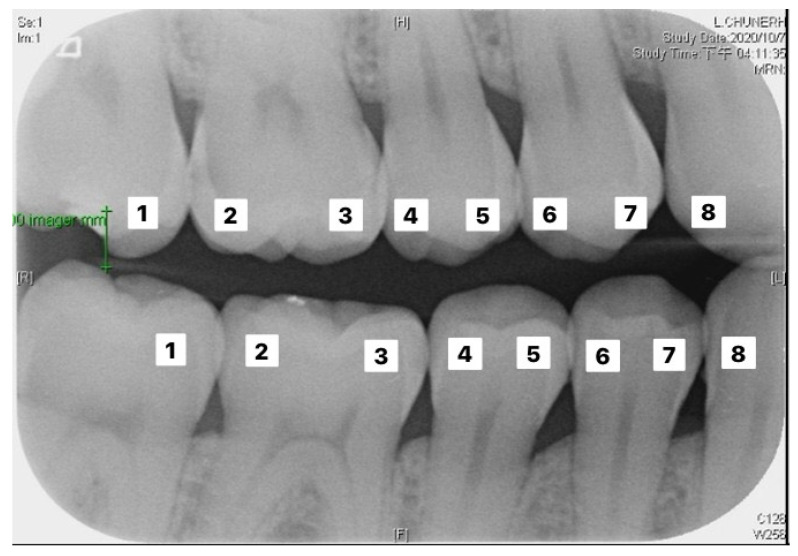
Image example of outer teeth for validation (from left to right, in order of 1–8).

The validation set was used to evaluate the performance of the two established models. The actual number of findings in the photo with the predicted number of findings in the photo were compared to calculate the accuracy. The formula of accuracy is shown in Equation (7). Table 4 and Table 5 correspond to the execution results of the upper and lower teeth in Figure 9, respectively.

**Figure 10:**
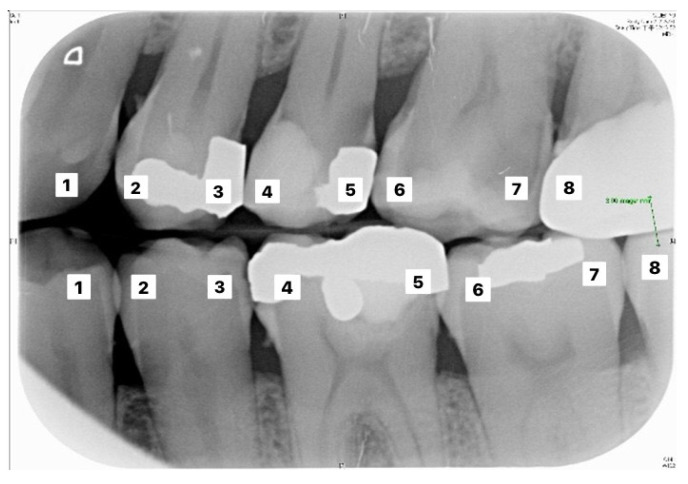
Image example of outer teeth for validation (from left to right, in order of 1–8).

Table 6 and Table 7 correspond to the judgment of the upper and lower rows of teeth in Figure 10. Results show that the accuracy of the proposed model for judging the restorations was 95.56%, which is an improvement compared to Lin et al. [30], with an accuracy of 90.23%. The proposed method in Lin et al. [30] first enhanced the classification features of the image, and then added the regular term and impulse, before establishing a CNN model with the ReLU function. With regard to judging caries, the proposed model in this study showed an accuracy of 90.30%, which was also an improvement compared to Singh and Sehgal [31], with an accuracy of 80.00%, which used a neural network classifier to classify caries. Table 8 and Table 9 are the truth tables of different CNN models.

(7) $$Accuracy=\frac{Correct\;predict\;images}{total\;images}\times 100\%.$$

There were two main reasons for the significant improvement in accuracy in this study. One involved masking some pixels in the photo to be classified which could cause confusion when training the CNN model and misjudgment when verifying the CNN model. This seemingly simple step had a significant impact on training and verifying the CNN model. The other reason could be attributed to the size of the photo used in training the CNN model. Having a very large size of input photo leads to the loss function not being able to converge, thus increasing the training time and reducing the accuracy. Therefore, this study reduced the image size to solve the abovementioned problem.

**Figure 11:**
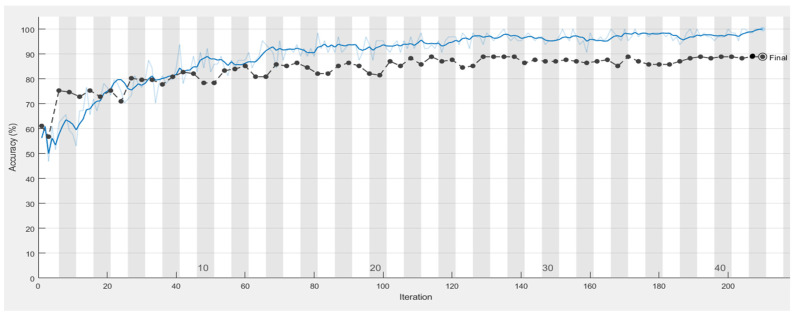
Training process of the model in classifying caries, with the blue line illustrating the accuracy of the training set.

**Figure 12:**
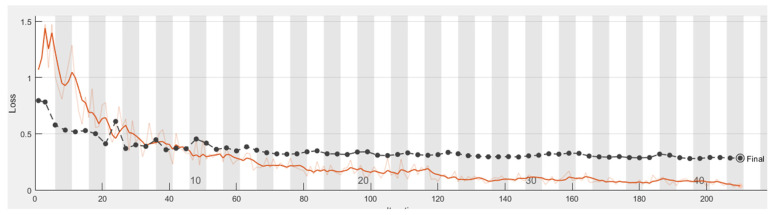
Loss process of the model in classifying caries, with the orange line illustrating the loss of the training set.

**Figure 13:**
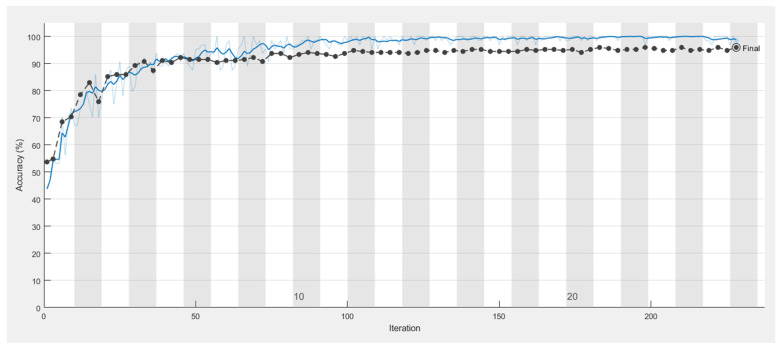
Training process of the model in classifying restorations, with the black and blue lines illustrating the accuracy of the test set and training set, respectively.

**Figure 14:**
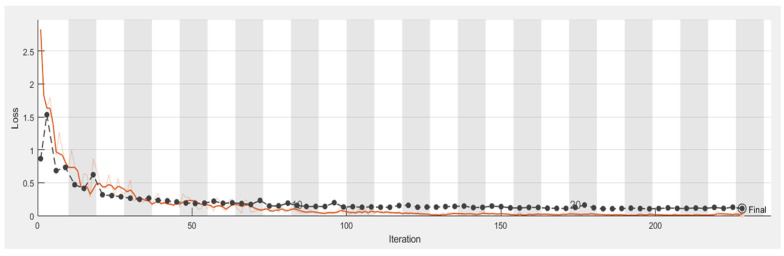
Loss process of the model in classifying restorations, with the black and orange lines illustrating the loss of the test set and training set, respectively.

It is shown from Figure 11, Figure 12, Figure 13 and Figure 14 that a smaller loss function led to higher accuracy of the reflection. As described in Section 2.4.1. GoogleNet, Vgg19, and ResNet50 were used in transfer learning for comparison with AlexNet with the same parameters, thus allowing a comparison of the accuracy and duration.

According to Table 10 and Table 11, the parameter MiniBatchSize of Vgg19 was different from the other networks, because Vgg19 needs a large capacity and, thus, more memory in the GPU.

Therefore, a larger BatchSize would hinder loading of the hardware, resulting in an inability to train; thus, reducing BatchSize here would facilitate the training. AlexNet’s elapsed time was the fastest among these networks. AlexNet was not the most accurate in the classification of restorations, but it could achieve excellent accuracy for both symptoms. Therefore, this paper chose it as the main network to classify the symptoms of disease. This study also compares the accuracy of different neural networks with the methods of reference papers Lin et al. [30] and Chen et al. [31], as shown in Table 12.

In this study, it was very complicated to determine whether there were restorations in the tooth in the photo. However, an improvement was needed in determining whether there were caries in the tooth under consideration in a given photo. There are two research directions in the future. One of them is to preprocess the photos used to train the CNN model and to verify the accuracy of the CNN model to better present the image of caries in the photo. The other research direction is to use methods more suitable for the classification of caries and restorations to improve accuracy.

## 4. Discussion

The results of the restorations model had an accuracy as high as 95.56%, while the accuracy in the judgment of caries was as high as 90.30%. These results represent a significant improvement over previously proposed methods in the literature [30,31]. These accuracies provided this study with the confidence to further extend model development for applied clinical medicine. First of all, the aim is to continuously improve the accuracy to a level that is acceptable for the model to be applied clinically. Secondly, the aim is to acquire more findings for judgment. Adding periodontology to the model database can diversify the signs of judgment. Thirdly, the aim is to adjust the output of the results in order for them to be displayed in real time on the dentist’s screen. Dentists that are currently carrying out the course of diagnosis and treatment can then immediately analyze the objective data to optimize the current process. Lastly, the proposed and developed technology will be submitted for patent application to protect ongoing research for its development, as well as intellectual property.

## 5. Conclusions

In this study, a preprocessing method of dental samples was presented, along with a segmentation method for separating individual tooth samples, as well as the training of a comprehensive model to identify and classify caries and restorations. According to the results of the experiment, the reported accuracy verified the success of the proposed method. This was achieved through a combination of a Gaussian high-pass filter and iterative threshold to enhance the quality of the binarization. This in turn enabled subsequent cropping as an important foundation. The reduction in impurities in the image samples improved the training quality of the proposed model.

On the other hand, the accuracy of the classification of restorations was higher than the accuracy of the classification of caries. This was due to the color difference in the lesion areas of caries, which was less evident as compared to that in restoration areas. One of the recommended future studies is to add new image samples to improve the accuracy of the classification of caries, as well as R-CNN for numbering [19]. This study can hopefully improve the accuracy of classification and further reduce the clinical time, thus enabling dentists to focus on treatment planning and clinical operations.

## Figures and Tables

**Table 1:** Number of bitewing findings for clinical application.

Quantity of Findings				
	Restorations	Caries	Normal	Total
Quantity	610	88	3018	3716

**Table 2:** AlexNet vs. new AlexNet layer comparison.

AlexNet Layer Activations			New Alexnet Layer Activations		
	Name	Activations		Name	Activations
1	Input	227 × 227 × 3	1	Input	200 × 100 × 3
2	Convolution	55 × 55 × 96	2	Convolution	48 × 23 × 96
3	Relu	55 × 55 × 96	3	Relu	48 × 23 × 96
4	Normalization	55 × 55 × 96	4	Normalization	48 × 23 × 96
5	Maxpooling	27 × 27 × 96	5	Maxpooling	23 × 11× 96
6	Convolution	27 × 27 × 256	6	Convolution	23 × 11 × 256
7	Relu	27 × 27 × 256	7	Relu	23 × 11 × 256
8	Normalization	27 × 27 × 256	8	Normalization	23 × 11 × 256
9	Maxpooling	13 × 13 × 256	9	Maxpooling	11 × 5 × 256
10	Convolution	13 × 13 × 384	10	Convolution	11 × 5 × 384
11	Relu	13 × 13 × 384	11	Relu	11 × 5 × 384
12	Convolution	13 × 13 × 384	12	Convolution	11 × 5 × 384
13	Relu	13 × 13 × 384	13	Relu	11 × 5 × 384
14	Convolution	13 × 13 × 256	14	Convolution	11 × 5 × 256
15	Relu	13 × 13 × 256	15	Relu	11 × 5 × 256
16	Maxpooling	6 × 6 × 256	16	Maxpooling	5 × 2 × 256
17	Fully-Connected	1 × 1 × 4096	17	Fully-Connected	1 × 1 × 1280
18	Relu	1 × 1 × 4096	18	Relu	1 × 1 × 1280
19	Dropout	1 × 1 × 4096	19	Dropout	1 × 1 × 1280
20	Fully-Connected	1 × 1 × 4096	20	Fully-Connected	1 × 1 × 1280
21	Relu	1 × 1 × 4096	21	Relu	1 × 1 × 1280
22	Dropout	1 × 1 × 4096	22	Dropout	1 × 1 × 1280
23	Fully-Connected	1 × 1 × 1000	23	Fully-Connected	1 × 1 × 2
24	Softmax	1 × 1 × 1000	24	Softmax	1 × 1 × 2
25	Classoutput	1000	25	Classoutput	2

**Table 3:** The hyperparameter settings used in this study.

Hyperparameters Used in This Study			
Momentum	LearnRate	Max Epochs	Mini BatchSize
0.9	0.00006	100	64

**Table 4:** Upper tooth judgement for the image in Figure 9.

Upper Tooth Judgement		
Number	Clinical Data	This Study
1	Normal	99.1% to be normal
2	Caries	99.9% to be caries
3	Normal	99.9% to be normal
4	Normal	95.9% to be normal
5	Normal	99.4% to be normal
6	Normal	96.7% to be normal
7	Normal	97.9% to be normal
8	Normal	99.5% to be normal

**Table 5:** Lower tooth judgement for the image in Figure 9.

Lower Tooth Judgement		
Number	Clinical Data	This Study
1	Normal	100.0% to be normal
2	Normal	94.9% to be normal
3	Normal	98.0% to be normal
4	Normal	99.9% to be normal
5	Normal	99.8% to be normal
6	Normal	99.9% to be normal
7	Normal	99.8% to be normal
8	Normal	99.1% to be normal

**Table 6:** Upper tooth judgement for teeth image in Figure 10.

Upper Tooth Judgement		
Number	Clinical Data	This Study
1	Normal	96.9% to be normal
2	Normal	96.9% to be normal
3	Caries Restorations	100.0% to be caries 99.8% to be restorations
4	Restorations	99.9% to be restorations
5	Caries Restorations	79.8% to be caries 99.7% to be restorations
6	Caries	96.8% to be caries
7	Caries	97.5% to be caries
8	Normal	96.9% to be normal

**Table 7:** Lower tooth judgement for the image in Figure 10.

Lower Tooth Judgement		
Number	Clinical Data	This Study
1	Normal	99.5% to be normal
2	Normal	98.6% to be normal
3	Restorations	99.7% to be restorations
4	Restorations	97.7% to be restorations
5	Normal	94.9% to be normal
6	Normal	93.6% to be normal
7	Normal	97.0% to be normal
8	Normal	99.7% to be normal

**Table 8:** Truth table of accuracy for classifying caries.

Accuracy of Classifying Caries		
Actual Predicted	True	False
True	85.55%	14.45%
False	5.33%	94.67%

**Table 9:** Truth table of accuracy for classifying restorations.

Accuracy of Classifying Restorations		
Actual Predicted	True	False
True	95.93%	4.07%
False	4.08%	95.92%

**Table 10:** Network comparison for caries.

Network Comparison for Caries				
	AlexNet	GoogleNet	Vgg19	ResNet50
Accuracy	90.30%	87.04%	80.25%	82.72%
Loss	0.2556	0.354	0.3821	0.407
MaxEpoch	100	100	100	100
MiniBatchSize	64	64	5	64
Iterations per epoch	5	5	75	5
Max iterations	500	500	7500	500
Validation patience	10	10	10	10
Learning rate	0.00006	0.00006	0.00006	0.00006
Elapsed time	1 min 34 s	5 min 44 s	6 min 32 s	106 min 4 s

**Table 11:** Network comparison for restorations.

Network Comparison for Restorations				
	AlexNet	GoogleNet	Vgg19	ResNet50
Accuracy	95.56%	98.44%	94.44%	96.67%
Loss	0.1134	0.068	0.1241	0.0979
MaxEpoch	100	100	100	100
MiniBatchSize	64	64	5	64
Iterations per epoch	9	9	63	9
Max iterations	900	900	6300	900
Validation patience	10	10	10	10
Learning rate	0.00006	0.00006	0.00006	0.00006
Elapsed time	2 min 6 s	9 min 47 s	14 min 9 s	64 min 35 s

**Table 12:** Comparison of accuracy with other papers.

Comparison of Accuracy with Other Papers						
	Our Method with Four Different Models of Transfer Learning				Method in [30]	Method in [31]
Used model	AlexNet	GoogleNet	Vgg19	ResNet	CNN	Neural Network Classifier
Accuracy in classifying caries	90.30%	87.04%	80.25%	82.72%		80.00%
Accuracy in classifying restorations	95.56%	98.44%	94.44%	96.67%	90.23%	
